# Krt5^+^/Krt15^+^ foregut basal progenitors give rise to cyclooxygenase-2-dependent tumours in response to gastric acid stress

**DOI:** 10.1038/s41467-019-10194-0

**Published:** 2019-05-20

**Authors:** Hyeongsun Moon, Jerry Zhu, Leanne R. Donahue, Eunju Choi, Andrew C. White

**Affiliations:** 1 Department of Biomedical Sciences, Cornell University, Ithaca, NY 14853 USA; 2 Department of Pathology, Microbiology and Immunology, University of California Davis, Davis, CA 95616 USA

**Keywords:** Cancer, Gastric cancer, Cell biology

## Abstract

The effective prevention of tumor initiation, especially for potentially inoperable tumors, will be beneficial to obtain an overall higher quality of our health and life. Hence, thorough understanding of the pathophysiological mechanisms of early tumor formation arising from identifiable cellular origins is required to develop efficient preventative and early treatment options for each tumor type. Here, using genetically engineered mouse models, we provide preclinical experimental evidence for a long-standing open question regarding the pathophysiological potential of a microenvironmental and physiological stressor in tumor development, gastric acid-mediated regional microscopic injury in foregut squamous epithelia. This study demonstrates the association of gastric acid stress with Cyclooxygenase-2-dependent tumor formation originating from tumor-competent Krt5^+^/Krt15^+^ foregut basal progenitor cells. Our findings suggest that clinical management of microenvironmental stressor-mediated microscopic injury may be important in delaying tumor initiation from foregut basal progenitor cells expressing pre-existing tumorigenic mutation(s) and genetic alteration(s).

## Abstract (web summary)

Cellular extrinsic environmental factors contribute to tumour development. Here, the authors show that gastric acid stress stimulates tumour formation from a defined tumour-competent Krt5 + /Krt15 + foregut basal progenitor cell population.

## Introduction

Cellular-extrinsic factors and their contribution to tumor development are of great interest in cancer etiology. As genetically engineered animal models have been widely developed and used to model biological processes such as cancer, the role of stem/progenitor cells with oncogenic mutation(s) and/or alteration(s) have been well described^1,2^. However, while stem/progenitor cells have often been shown to act as cells of origin in various types of cancers^1^, little is known regarding the contribution of cellular extrinsic factors such as environmental and physiological stressors in tumor formation from tumor-prone stem/progenitor cells.

Importantly, a recent study that analyzed epidemiological and genetic data proposed a mathematical hypothesis regarding the correlation between the lifetime risk of cancers and stem cell division rate^3^. It is also important to note that although stem cell division rate appears to play a crucial role in the development of driver mutations for tumors, cellular extrinsic stressors can often prove to be the tipping point for whether or not genetically prone cells become tumorigenic^4,5^. In addition to these mathematical hypotheses, several lines of experimental evidence support the hypotheses that preventing stress factors could potentially reduce the lifetime risk of cancer^6–8^. For instance, as an environmental stressor, ultraviolet light can significantly promote the formation of cutaneous melanoma from tumor-prone melanocyte stem cells in the skin^6,7^, and bacterial prostatitis can stimulate prostate cancer development from basal progenitors in the prostate^8^.

Significantly, esophageal cancer is one of the most frequent causes of cancer-related deaths worldwide. Its 5-year survival rate of <20% has not improved in the past several decades^9–12^. Since there are significant limitations in the ability to detect tumors from the esophagus at early asymptomatic stages, over 50% of patients have inoperable tumors or detectable metastases at diagnosis^12–14^. Despite a wealth of epidemiological data and knowledge regarding potential risk factors for esophageal cancer, the role of microenvironmental conditions that can modulate cellular behavior and tumor development remains poorly understood. Among various physiological conditions, gastroesophageal reflux may have a significant impact on esophageal cancer development and progression as a primary or co-promoting risk factor^15^. The pathological contribution of gastroesophageal reflux disease (GERD) has been relatively well described in the progression of Barrett’s metaplasia toward esophageal adenocarcinoma^16,17^. However, the role of GERD in esophageal squamous cell carcinoma (ESCC) remains unclear^15^. Therefore, using genetically engineered mouse models, we investigated if microenvironmental gastric acid stress can act as a co-promoting factor, which may significantly accelerate early tumor formation from foregut squamous epithelia, particularly from mutant long-lived basal progenitor cells expressing a pre-existing oncogenic load.

The murine forestomach is a continuation of the stratified epithelium of the esophagus and can be considered a dilated form of the lower esophageal tract. Here, we demonstrate that Keratin5^+^ and Keratin15^+^ (Krt5^+^, Krt15^+^) basal progenitors^18^ of the forestomach have high susceptibility to oncogenic Ras-mediated tumor formation when exposed to regional, microenvironmental gastric acid stress. Furthermore, the data presented show that tumor-competent basal progenitors differentiate normally or show mild histological hyperplasia when physiological gastric acid stress is suppressed by treatment with proton pump inhibitors (PPIs). This study further reveals that gastric acid stress-induced acceleration of tumor formation from mutant foregut basal progenitors is dependent on epithelial-specific prostaglandin-endoperoxide synthase 2 (Ptgs2, also known as cyclooxygenase-2, Cox-2) expression. Taken together, this study demonstrates the significant contribution of physiological acid stress as a co-promoting factor in foregut tumor initiation from tumor-competent Krt5^+^/Krt15^+^ basal progenitor cells.

## Results

### Higher tumor susceptibility at the region near the SCJ

The long-term lifespan of stem/progenitor cells can allow these cells to accumulate more mutations than terminally differentiated, short-lived cells^1^. It has been reported that Krt5^+^ and Sox2^+^ basal progenitors can act as the cellular origin for murine foregut tumor formation^19,20^. Unlike p53, mutations are not frequently found in Kras or Pten; however, enhanced RTK/Ras/PI3K/Akt signaling and p53 mutations are characteristic of altered signaling pathways found in human ESCC and are hypothesized to be sufficient to induce murine foregut tumor formation from basal progenitors^20,21^. To recapitulate the molecular signatures of ESCC in human patients, we utilized genetic alleles for conditional knock-in of Kras^G12D^ (cell-type-specific, constitutively active form of Kras)^22^ or knockout of p53^23^ or Pten^24^ (cell-type-specific, p53 or Pten loss of function).

**Fig. 1:**
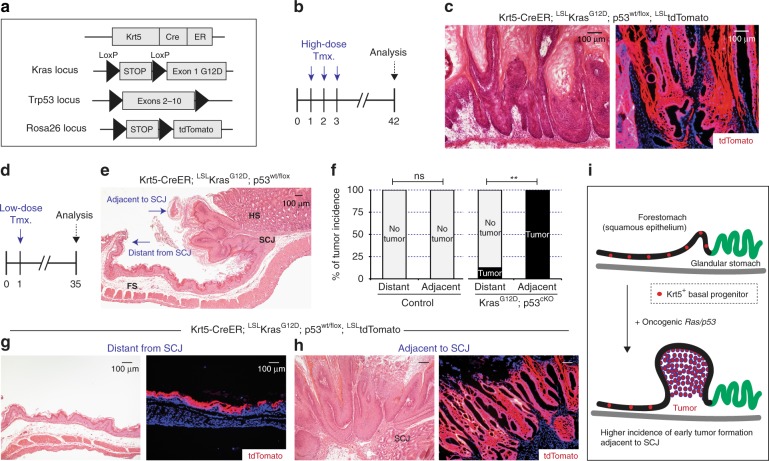
Higher susceptibility of tumor initiation from Keratin5^+^ (Krt5^+^) progenitors near the SCJ. **a**, **b** Experimental scheme. **c** Oncogenic Ras/p53-mediated tumor formation throughout the forestomach tissues. **d** Experimental scheme. **e**, **f** Histological phenotype of early tumor initiation and higher tumor incidence from tumor-prone Krt5^+^ progenitors resident at regions adjacent to the SCJ (*n* = 8 animals per each). Statistical significance was determined by Fisher’s exact test; ns = not significant, **p < 0.005. **g**, **h** Histological phenotype of early tumor initiation and tdTomato lineage tracing. **i** Summary of early tumor formation from tumor-prone Krt5^+^ basal progenitors expressing oncogenic Ras/p53 combination. FS, forestomach; HS, hindstomach; SCJ, squamocolumnar junction; Tmx, tamoxifen. Scale bars, 100 µm

To understand the early steps of tumor initiation from foregut basal progenitors, we generated Krt5-CreER; LSL-Kras^G12D^; p53^wt/flox^; LSL-tdTomato mice^22,23,25,26^. The Krt5-CreER genetic allele can label foregut basal progenitors based on tamoxifen-inducible Cre recombinase expression^26^. Thus, systemic treatment of tamoxifen by intraperitoneal (i.p.) injection in our adult mice induced the cell-type-specific expression of oncogenic Kras^G12D^ together with tumor suppressor p53 loss of function (hereafter oncogenic Ras/p53 combination). As previous studies suggest^19,20^, the oncogenic Ras/p53 combination mediated aggressive development of squamous papillomas 50 days following 3 consecutive days of 2 mg tamoxifen treatment by i.p. injection. Squamous papillomas are considered a precursor lesion that have the potential to evolve into a squamous cell carcinoma, which were observed throughout the entire forestomach tissue (Fig. 1a–c). In our mice, we also used a Loxp-Stop-Loxp-tdTomato (LSL-tdTomato) genetic allele^25^ for lineage tracing of our tumor-prone progenitors. tdTomato expression indicated that tumor cells originated from Krt5^+^ basal progenitors expressing the oncogenic Ras/p53 combination rather than neighboring Krt5^−^ cell populations (Fig. 1c).

The significantly higher tumor incidence within the forestomach tissues (Fig. 1c) compared to the esophageal lumen is potentially due to a difference in the acidic microenvironment^19^. Therefore, to characterize early tumorigenesis from tumor-prone Krt5^+^ foregut basal progenitors and to determine their relative tumor susceptibility, we used low-dose tamoxifen treatment (1 day of 0.2 mg tamoxifen by i.p. injection) and analyzed the results (Fig. 1d). Intriguingly, squamous tumor susceptibility within the forestomach tissues was significantly higher from tumor-competent basal progenitors resident at regions adjacent to the squamocolumnar junction (SCJ), as compared to regions distant from the SCJ (Fig. 1d–i). This brought our attention to the potential contribution of microenvironmental regional stress in increasing tumor susceptibility from tumor-competent basal progenitors (Fig. 1d–i).

### Higher tumorigenesis from SCJ-adjacent Krt15^+^ progenitors

Recently, the Krt15-CrePR transgene has been shown to target esophageal basal progenitors involved in tissue homeostasis in response to radioactive injuries^18^. We also observed that basal progenitors within the murine forestomach tissues express both Krt5 and Krt15 (Supplementary Fig. 1a). Since murine foregut tumor formation induced by oncogenic Ras/p53 expression is more frequently found in the region adjacent to the SCJ within the forestomach tissues (Fig. 1), we investigated whether the Krt15-CrePR transgene^27^ could selectively label progenitor cells within the forestomach (squamous epithelia), but not cells within the hindstomach tissues (glandular epithelia).

**Fig. 2:**
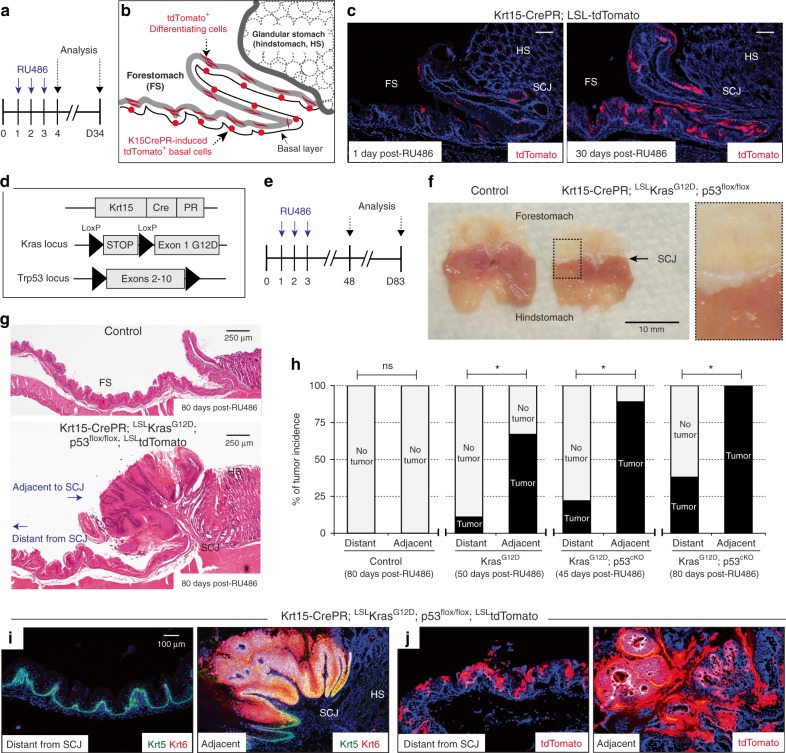
Higher susceptibility of tumor development from Keratin15^+^ (Krt15^+^) progenitors near the SCJ. **a** Experimental scheme. **b**, **c** tdTomato-positive Krt15^+^ progenitors and their differentiated daughter cells, scale bar 100 µm. **d**, **e** Experimental scheme. **f**, **g** Macroscopic and histological phenotype of Ras/p53-mediated tumor formation throughout the regions adjacent to SCJ. Scale bars, 250 µm. **h** Tumor incidence from tumor-competent Krt15^+^ progenitors. Control, *n* = 9 animals. Krt15-CrePR; LSL-Kras^G12D^, *n* = 9 animals. Krt15-CrePR; LSL-Kras^G12D^; p53^flox/flox^ (45 days, *n* = 9 animals; 80 days, *n* = 8 animals). Statistical significance was determined by Fisher’s exact test; ns = not significant, *p < 0.05. **i**, **j** Immunostaining for Krt5 and Krt6, and tdTomato lineage tracing demonstrated significant tumor formation from tumor-competent Krt15-CrePR+ progenitors resident at the region adjacent to SCJ. Scale bar, 100 µm. FS, forestomach; HS, hindstomach; SCJ, squamocolumnar junction

To induce Cre recombinase expression in Krt15-CrePR mice, we used RU486 that can bind to progesterone receptor (PR) fusion protein (Fig. 2a). Then, using LSL-tdTomato-mediated lineage tracing^25^, we confirmed that Krt15-CrePR can selectively target basal progenitors within forestomach tissues, but not other cells in the hindstomach (Fig. 2b, c). Importantly, the lack of hindstomach expression eliminates the possibility of confounding field effects in tumorigenesis experiments. Furthermore, as expected from a previous report^18^, tdTomato-positive differentiated cells originating from Krt15^+^ basal progenitors labeled by Krt15-CrePR were observed over time indicating their continuous contribution as a long-lived basal progenitor (Fig. 2b, c).

Compared to Krt5-CreER with low-dose tamoxifen treatment, the Krt15-CrePR transgene (1 day after 3 consecutive days of 2 mg RU486 administration) labeled a significantly limited number of basal progenitors within the forestomach (Supplementary Fig. 1b, c), which allowed us to examine the early steps of tumor formation arising from fewer total cells undergoing recombination. In addition, since conditional expression of oncogenic Kras mutation using the LSL-Kras^G12D^ genetic allele can cause significant health concerns in Krt5-CreER mice due to tumor susceptibility throughout the body (i.e., significant hyperplasia from oral and palm tissues), using the Krt15-CrePR genetic allele may minimize this experimental limitation.

To determine the tumor susceptibility of esophageal and forestomach Krt15^+^ progenitor cells, we introduced oncogenic Kras^G12D^ expression together with loss of p53 function (Fig. 2d, e) in Krt15^+^ cells. Similar to progenitors labeled by Krt5-CreER, mice expressing these mutant alleles (LSL-Kras^G12D^ with/without p53^flox/flox^)^22,23^, showed high susceptibility to foregut tumor formation from Krt15^+^ progenitors at regions adjacent to the SCJ (Fig. 2f–j), as compared to regions distant from the SCJ. Keratin 6 (Krt6), a marker for hyperplastic epithelium, was distinctively expressed in tumorigenic regions adjacent to the SCJ (Fig. 2i). Furthermore, tdTomato lineage tracing revealed that tumor cells preferentially arise from the Krt15^+^ cells located in this adjacent region (Fig. 2j, Supplementary Fig. 2).

**Fig. 3:**
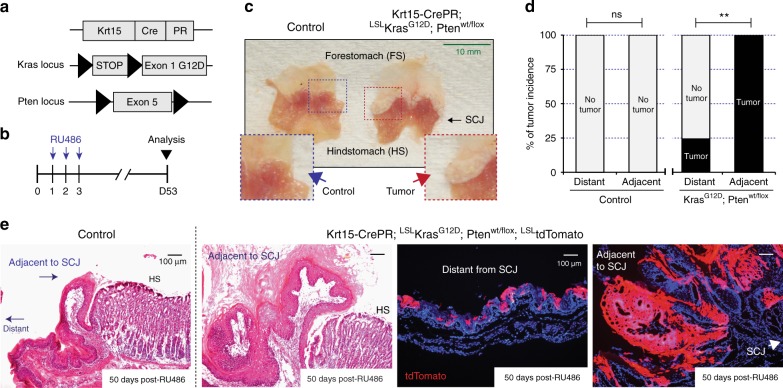
Higher Ras/Pten-induced tumor incidence at the region adjacent to the squamocolumnar junction (SCJ). **a**, **b** Experimental scheme. **c** Macroscopic phenotype indicates significant tumor formation throughout the region adjacent to the SCJ. **d** Tumor incidence from tumor-prone Keratin15^+^ (Krt15^+^) progenitors. Control, *n* = 7 animals. Krt15-CrePR; LSL-Kras^G12D^; Pten^wt/flox^, *n* = 8 animals. Statistical significance was determined by Fisher’s exact test; ns = not significant, **p < 0.005. **e** Histology and tdTomato lineage tracing revealed tumors from tumor-prone Krt15^+^ progenitors adjacent to the SCJ, while distant progenitors do not show aberrant growth. Scale bars, 100 µm

To identify whether the higher tumor incidence at the SCJ-adjacent region is specific to the oncogenic Ras/p53 combination, we substituted p53 loss for another tumor suppressor, Pten, using a conditional knockout allele for Pten^24^ (hereafter oncogenic Ras/Pten) (Fig. 3a, b). Intriguingly, in Krt15-CrePR; LSL-Kras^G12D^; Pten^wt/flox^; LSL-tdTomato mice, tumor growth significantly increased in the context of Pten loss of function; however, the incidence of oncogenic Ras/Pten-mediated foregut tumor formation was consistently higher from Krt15^+^ basal progenitors resident to the region adjacent to the SCJ, consistent with the p53 loss model (Fig. 3c–e, Supplementary Fig. 3).

One possible explanation for higher tumorigenic preference at the adjacent region is simply due to an increase in the number of tumor-prone cells expressing both oncogenic Ras/p53 and Ras/Pten. To test this, the total number of recombined cells, as determined by lineage tracing marker tdTomato expression, was quantified in both the SCJ-adjacent and SCJ-distant regions. However, the total number of recombined cells was found to be similar between regions adjacent to and distant from the SCJ (Supplementary Fig. 4a, b).

The mathematical hypothesis of stem cell originating cancers indicates that tissues harboring cells with a higher cell division rate have a higher incidence of cancer^3^. To explore if there was a difference in division rate at regions adjacent to and distant from the SCJ, the Ki-67 proliferation index was measured in wild-type cells. Intriguingly, while the overall number of tdTomato-positive progenitors was similar between distant and adjacent regions, the proliferation index was significantly higher at the region adjacent to the SCJ (Supplementary Fig. 4c, d) compared to more distant regions.

It is known that while the forestomach and hindstomach share the same luminal space, regional pH differences were noted between the forestomach (pH 4.8 ± 0.3) and the hindstomach (pH 3.0 ± 0.4)^28^. Furthermore, we found that in healthy wild-type control mice, forestomach epithelia near the SCJ express a marker of hyperplastic epithelia, Krt6^29^, which suggests prolonged microenvironmental stress from regional gastric acid (Supplementary Fig. 5a). Moreover, we examined if recently identified Krt7^+^ subpopulations at the SCJ^30^ are significantly associated with higher tumor susceptibility. As previously reported^30^, we confirmed the presence of both Krt5^+^/Krt7^+^ basal progenitors and Krt5^−^/Krt7^+^ luminal cells at the SCJ (Supplementary Fig. 5a). However, based on our tissue staining, significant tumorigenic contribution was not detected from both Krt7^+^ progenitors and luminal cells during early foregut tumor formation from squamous epithelia (Supplementary Fig. 5). More likely, squamous epithelium expressing both Krt5 and Krt6 adjacent to the SCJ, which has been previously defined^31^, appeared to be the source of early tumor cells (Supplementary Fig. 5). Early hyperplastic tumor cells showed high expression of Krt6 and Krt5, decreased expression of Loricrin (Lor), and a lack of Krt7 expression (Supplementary Fig. 5b). On the other hand, Krt7^+^ basal progenitors and luminal cells appeared to remain intact at the SCJ, with no apparent expansion (Supplementary Fig. 5b).

### Influence of gastric acid stress in tumor susceptibility

The observed difference in division rate and persistent Krt6 expression in regions of squamous epithelia adjacent to the SCJ (Supplementary Figs. 4 and 5) may have an important influence on tumor development susceptibility. The differences could be caused by intrinsic cellular properties at these two regions. Alternatively, differences may be due to environmental stressors from the gastric hindstomach, which could stimulate tissue turnover at regions adjacent to the SCJ. Although the Krt7^+^ subpopulation did not appear to contribute to foregut tumor formation (Supplementary Fig. 5), it is still possible that there is an unknown subpopulation that may have significantly higher tumor susceptibility.

**Fig. 4:**
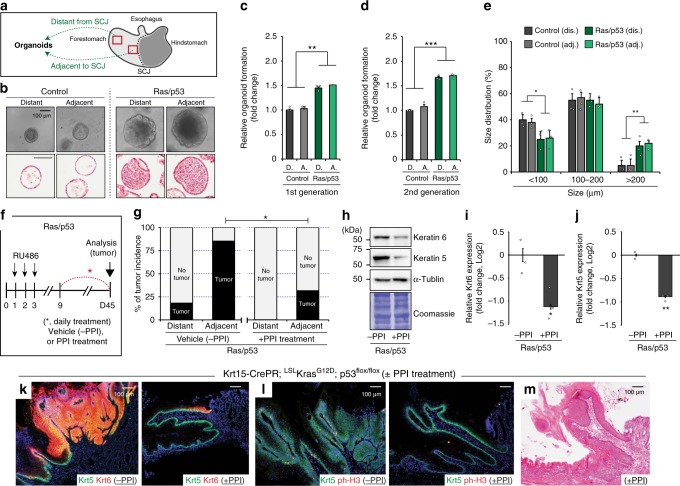
Microenvironmental acidic stressors and tumor susceptibility. **a** Experimental scheme. Epithelial cells isolated from the regions adjacent or distant from squamocolumnar junction (SCJ). **b** Microscopic phenotype of three-dimensional (3D) organoids from control and Krt15-CrePR; LSL-Kras^G12D^; p53^flox/flox^ mice. Histology of 3D organoids demonstrated that oncogenic Ras/p53 increased abnormal growth features. **c**–**e** Quantification of relative organoid formation (*n* = 3 independent experiments, 25 fields at high power field (HPF) per sample) and size distribution (*n* ≥ 150) from control and experimental mice expressing oncogenic Ras/p53 combination. **f** Experimental scheme. Phosphate-buffered saline (PBS) (vehicle) or PPI were daily treated by intraperitoneal (i.p.) injections (5 consecutive days per week). **g** Tumor incidence from Krt15-CrePR; LSL-Kras^G12D^; p53^flox/flox^ mice, with/without PPI treatment, *n* = 9 animals per each. Statistical significance was determined by Fisher’s exact test; *p < 0.05. **h**–**j** Immunoblotting of Keratin5 (Krt5) and Krt6 demonstrated relatively less tumor formation in the PPI treatment group, *n* = 5 independent experiments. **k**–**m** Immunostaining of Krt5, Krt6, and phospho-histone H3 (ph-H3), and histology demonstrated suppressed tumor formation by daily PPI treatment in Krt15-CrePR; LSL-Kras^G12D^; p53^flox/flox^ mice. PPI, proton pump inhibitor. Data with bar graphs are represented as mean ± SEM. Statistical significance was determined by pair-wise comparison using *t* test; *p < 0.05, **p < 0.005. Scale bars, 100 µm

In order to determine if Krt15^+^ cells at SCJ-adjacent and SCJ-distant regions had intrinsic differences in tumor susceptibility, we utilized the three-dimensional (3D) organoid system schematized in Fig. 4a. While the expression of Kras^G12D^ together with loss of p53 function significantly increased organoid formation efficiency and size, there appeared to be no distinction in formation and size between the adjacent and distant regions (Fig. 4b–e). However, while organoids from control tissue demonstrated a hollow lumen, organoids derived from Ras/p53 cells showed hypercellularity that caused these lumens to be filled by multinucleated cells (Fig. 4b). These data indicate that oncogenic Krt15^+^ cells from SCJ-adjacent and SCJ-distant regions do not have differing intrinsic propensities for tumor formation.

These organoid experiments suggest that the tumorigenic potential from Krt15^+^ progenitors between SCJ-adjacent and SCJ-distant regions is similar. Alternatively, we hypothesized that the regional microenvironment may significantly influence tumor initiation from tumor-competent Krt15^+^ progenitors. In this case, increased stress and tissue turnover in the SCJ-distant regions should heighten tumor formation. To test this hypothesis, we administered low-pH acidified (≤pH 3) drinking water and compared regional tumor incidence to control animals supplied with normal drinking water. Interestingly, tumor incidence showed an upward trend in the number of tumors in regions distant from the SCJ in animals given acidified drinking water (Supplementary Fig. 6a–d).

Data provided by these acidified water experiments suggested the converse experiment that reduced gastric acid-induced stress could inhibit tumor development. To suppress microenvironmental gastric acid stress, a PPI, pantoprazole, was administered to tumor-prone and control animals (Fig. 4f). Daily treatment with pantoprazole significantly decreased tumor incidence (Fig. 4g–m). Pantoprazole suppressed the hyperplastic signature indicated by Krt6 expression, increased levels of Krt5 (a marker of tumors originating from squamous epithelia) (Fig. 4h–k), and reduced epithelial proliferation, as indicated by phospho-histone H3 (ph-H3) antibody staining (Fig. 4l).

Regional tissue microscopic injury is one of the hallmarks of tumor initiation, and chronic irritation is known to be a key factor influencing ESCC development^32,33^. In our mouse model, one notable well-observed histological phenotype was an increased inflammation burden during tumor formation at the SCJ-adjacent region, and expression of inflammatory markers following PPI treatment was decreased (Supplementary Fig. 7). These observations indicate that injury- and stress-induced inflammation due to the low-pH environment could be important factors facilitating tumor development in regions adjacent to the SCJ.

### Cell-type-specific Cox-2-dependent tumor formation

**Fig. 5:**
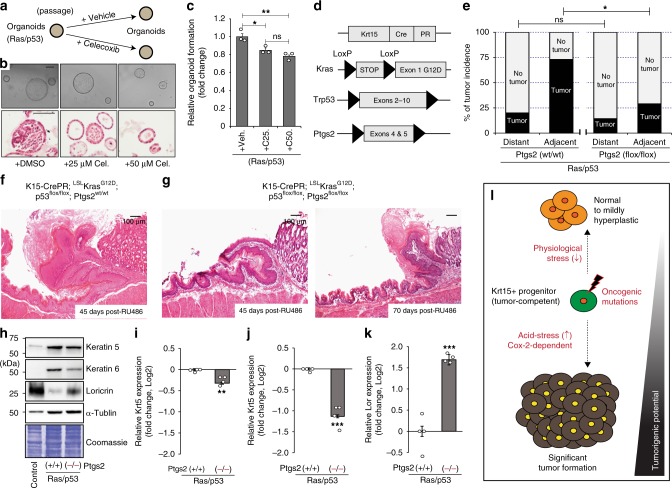
Role of cell-type-specific cyclooxygenase-2 (Cox-2) expression in Ras/p53-mediated tumor formation. **a** Experimental scheme. **b** Microscopic phenotype and histology of three-dimensional (3D) organoids with/without celecoxib treatment. **c** Relative 3D organoid formation was determined by the number of organoids larger than 100 µm, *n* = 3 independent experiments, 25 fields at high power field (HPF) per sample. Veh, vehicle control; C25, 25 µM; C50, 50 µM celecoxib. **d** Experimental scheme. **e** Ras/p53-mediated tumor incidence with/without condition Cox-2 knockout. Ptgs2^wt/wt^, *n* = 15 animals; Ptgs2^flox/flox^, *n* = 14 animals. **f**, **g** Histological phenotypes. **h**–**k** Immunoblotting of Keratin5 (Krt5), Krt6 and Loricrin (Lor) demonstrated decreased tumor susceptibility but increased differentiation status, *n* = 4 independent experiments. **l** Summary of the contribution of physiological stress factors in the susceptibility of tumor development from tumor-competent Krt15^+^ progenitors. Data with bar graphs are represented as mean ± SEM. Statistical significance was determined by pair-wise comparison using *t* test; ns = not significant, *p < 0.05, **p < 0.005. Scale bars, 100 µm. Ptgs2 prostaglandin-endoperoxide synthase 2

Gastric acid stress on the esophageal mucosa could result in inflammation via activation of Cox-2, nuclear factor-κB (NF-κB), and sterol regulatory element-binding protein (SREBP) pathways, which are frequently upregulated in cancer cells^34–36^. Cox-2 is an especially well-known pro-inflammatory mediator that is commonly observed in human ESCC patients^37–42^. Since it has also been reported that low-pH can significantly induce the activation of pathways related to NF-κB and Cox-2 in the esophageal tissues^34,35^, we determined if Cox-2 could be an underlying mediator of inflammation and foregut tumor development. Also, significant Cox-2 expression was observed in hyperplastic SCJ-adjacent tissues during tumor development (Supplementary Fig. 8a, b). To determine whether suppression of endogenous Cox-2 could reduce oncogenic Ras/p53-mediated tumor initiation, we treated 3D organoids with the selective Cox-2 inhibitor celecoxib (Fig. 5a). Earlier studies demonstrated that oncogenic Ras/p53 is sufficient to increase organoid formation and organoids often showed abnormal growth features (Fig. 4b). Celecoxib addition to stem cell media suppressed the ability to form organoids and notably reduced the hypercellularity found within the central organoid region (Fig. 5b, c), indicating that Cox-2 expression may be required for tumor formation in vivo.

To define the role of Cox-2 in oncogenic Ras/p53-mediated tumor formation arising from Krt15^+^ progenitors in vivo, we bred our mouse model to a conditional knockout allele for Ptgs2 (Cox-2)^43^ to induce Cox-2 loss of function in our tumor-prone progenitors (Fig. 5d). While oncogenic Ras/p53 expression significantly induced tumor formation from Krt15^+^ cells within the SCJ-adjacent region, loss of Ptgs2 function in Krt15^+^ cells significantly suppressed tumor development from tumor-competent Krt15^+^ basal progenitors (Fig. 5e–g). Consistent with known functions of Cox-2 enzymatic activity, Cox-2-knockout tissue demonstrated a decreased inflammation burden (Supplementary Fig. 8c). Finally, in addition to suppressed tumor formation (Fig. 5e–j), epithelial-specific Cox-2 deletion increased cellular differentiation, as indicated by significantly higher expression of Lor, a marker for terminally differentiated cells (Fig. 5h, k).

Taken together, Krt15^+^ foregut progenitors expressing a Ras/p53 tumorigenic load are capable of tumor development. However, initiation from these progenitors can be significantly accelerated by environmental gastric acid stress. Tumorigenesis from Krt15^+^ foregut progenitors requires squamous epithelial-specific Cox-2 expression for efficient tumor formation (Fig. 5l). These results further show that inhibition of cellular extrinsic stress factors to the foregut epithelia, in this case through PPI use, can suppress the process of tumor formation from tumor-competent Krt15^+^ progenitors and may increase the latency of tumor initiation (Fig. 5l). Together with maintenance of physiological stressors, inhibition of cellular intrinsic Cox-2 pathways may significantly delay tumor initiation from tumor-competent long-lived basal progenitors (Fig. 5l).

## Discussion

Esophageal cancer is one of the most lethal cancers worldwide and ESCC accounts for around 90% of esophageal cancer-related death^9–12,44^. To date, the most well-known risk factors include smoking and alcohol consumption^33^. Both smoking and alcohol lead to DNA mutations, and the combination of both risk factors is known to further increase the incidence rate of ESCC^45,46^. However, whether additional environmental and physiological stress factors can potentially contribute as co-promoting factors has not been studied. These stress factors may facilitate esophageal tumor initiation from cancer cells of origin expressing pre-existing genetic mutations and/or alterations known to be sufficient for ESCC development. The current studies aimed to understand the early steps of tumor initiation from basal progenitors containing a pre-existing mutational load, oncogenic Ras/p53 or Ras/Pten, and how the low-pH microenvironment may influence tumor initiation.

From our genetically engineered mouse model system, we speculated that physiological stressors such as reduced pH on tumor-competent basal progenitors may play an important role as a co-promoting factor in tumorigenesis. ESCC is most prevalent throughout Eastern Asia, and to a lesser extent, Eastern Europe and Africa^33,44^. Historically, GERD is less frequent; however, it is now evident that GERD is rapidly increasing in these Eastern Asian countries^47^. Smoking and/or alcohol consumption can also induce or increase the symptoms of GERD in human patients^48,49^. GERD is known to deliver both gastric and bile acids to the esophageal lumen, and it has been shown that bile acids, through duodenoesophageal reflux, can act as a mutagen, particularly for Barrett’s esophagus^33^. The mutagenicity of gastric acid has been unclear; hence, its contribution has been likely underappreciated within the upper gastrointestinal tract and early ESCC development. We were particularly interested in the role of gastric acid exposure as a microenvironmental stress factor acting as a co-promoting risk factor during tumor initiation from squamous epithelia, especially from foregut basal progenitors of the forestomach and esophagus.

Chronic exposure of gastric acid can enhance inflammatory pathways including Cox-2, NF-κB, and SREBP pathways, which are frequently upregulated in cancer cells^34–36^. In addition to the relationship between gastric acid and Cox-2 upregulation, extracellular acidic pH can also directly stimulate SREBP pathways leading to Cox-2 upregulation in the esophageal mucosa^36^. In addition to the direct effects on anti-gastric acid secretion, PPIs may have other beneficial effects including anti-oxidative stress and anti-inflammatory effects^50^. For instance, PPI treatment is known to beneficially control esophagitis and provide a significant reduction of Cox-1 and Cox-2 expression^51^. Our experimental results using genetically engineered mice further demonstrate the potential benefit of PPI treatment in tumor initiation from tumor-competent Krt15^+^ foregut basal progenitors. These results suggest the importance of clinical management of additional stress factors in patients who have been exposed to well-known risk factors for ESCC, such as long-term smokers with persistent GERD.

In summary, our study provides experimental evidence, which demonstrates the significant contribution of additional cellular-extrinsic stress factors in early tumor formation from tumor-competent long-lived foregut basal progenitors. We identified gastric acid as an important physiological stressor capable of initiating foregut tumorigenesis via a cell-type-specific Cox-2-dependent process. Moreover, Cox-2 loss of function can increase differentiation status, which can have a suppressive role in oncogene-mediated tumor formation. Taken together, these results provide a meaningful preclinical message that includes the importance of clinical maintenance of multiple stress factors for efficient tumor prevention, which may benefit human patients with heightened risk for ESCC.

## Methods

### Animals

Mice were acquired from Jackson Labs (Krt5-CreER, Krt15-CrePR, LSL-tdTomato, Pten^flox/flox^ and Ptgs2^flox/flox^) or the National Cancer Institute Mouse Models of Human Cancers Consortium repository (p53^flox/flox^ and LSL-Kras^G12D^). All animals were properly maintained under conditions set forth by the Institutional Animal Care and Use Committee (IACUC) and the Animal Research Committee at Cornell University. All animal protocols including ethical regulations were approved by the IACUC at Cornell University, before we conducted any experiments using animals. When mice were around 7 weeks postnatal, Krt5-CreER; LSL-Kras^G12D^; p53^wt/flox^; LSL-tdTomato, Krt15-CrePR; LSL-Kras^G12D^; LSL-tdTomato, Krt15-CrePR; LSL-Kras^G12D^, Krt15-CrePR; LSL-Kras^G12D^; p53^flox/flox^; LSL-tdTomato, Krt15-CrePR; LSL-Kras^G12D^; Pten^wt/flox^; LSL-tdTomato, and Krt15-CrePR; LSL-Kras^G12D^; p53^flox/flox^; Ptgs2^flox/flox^ animals were systemically treated by i.p. injections of tamoxifen or RU486 (mifepristone) as indicated in each result with experimental schemes. Tamoxifen and RU486 were dissolved in corn oil as 10 mg mL^−1^, then 200 µL per day was injected. For low-dose tamoxifen treatment, tamoxifen was dissolved in corn oil as 1 mg mL^−1^, then 200 µL per day was injected. For PPI treatment, mice were treated with pantoprazole (dissolved in phosphate-buffered saline (PBS), 10 mg kg^−1^ i.p., 5 days per week), and vehicle control group mice were treated with PBS by i.p. injections. Phenotypes shown from these mice were examined as indicated in each experimental scheme.

### Tissue immunostaining

OCT-embedded frozen (8 µm), or formalin-fixed and paraffin-embedded (5 µm) tissue sections were used for histology and immunostaining. Immunofluorescence staining and immunohistochemistry were carried out with antibodies described below. For formalin-fixed tissues, antigen retrieval was performed with citrate buffer at 92 °C for 30 min. Antibodies against Ki-67 (#14-5698, 1:300) were obtained from eBioscience, ph-H3 (#3377, 1:400) from Cell Signaling, Cox-2 (#160106, 1:200) from Cayman Chemical, Krt7 (#ab181598, 1:600) from Abcam, and Krt5 (#PRB-160P and # SIG-3475, 1:600), Krt6 (#PRB-169P, 1:600), Krt15 (#PCK-153P, 1:400), CD324 (#147306, 1:300), CD45 (#103101, 1:400), Gr-1 (#108417, 1:400), Ly-6G (#127625, 1:400), F4/80 (#123101, 1:600), and CD3 (#100201, 1:400) from BioLegend.

### Immunoblotting

Tissue lysates were collected using whole cell lysis buffer together with a homogenizer. Whole cell lysis buffer contains 20 mM Tris, pH 7.5, 150 mM NaCl, 1% Triton X-100, 10 mM sodium fluoride, 0.5 mM sodium vanadate, 1 mM sodium pyrophosphate, 0.5 mM 4-(2-aminmoethyl) benzensulfonyl fluoride hydrochloride and protease inhibitors, and protease inhibitors were freshly added into the whole cell lysis buffer immediately prior to protein collection^52^. Total protein concentration of tissue lysates was determined using bicinchoninic acid assay and a microplate reader following the manufacturer’s instructions. The same amount of tissue lysates denatured at 95 °C for 5 min were resolved on sodium dodecyl sulfate-polyacrylamide gel electrophoresis gels, and then transferred onto polyvinylidene difluoride (PVDF) membranes. Blocking was performed with 5% milk. PVDF membranes were incubated with primary antibodies. Antibodies against Krt5 (#PRB-160P, 1:2000), Krt6 (#PRB-169P, 1:2000), and Lor (#905104, 1:1000) were obtained from BioLegend, and α-tubulin (#NB600-506, 1:5000) from Novus. The signal was detected using peroxidase-conjugated secondary antibodies and an enhanced chemiluminescent horseradish peroxidase substrate. The intensity of signal was determined using ImageJ.

### 3D organoid culture

Epithelial cell isolation and 3D organoid culture were performed as recently defined^18,53^. Epithelial cells were isolated from mouse forestomach tissues, and then 20,000 cells were seeded in 100 µL Matrigel in 24-well plates. After Matrigel solidification, stem cell medium was added into the well and replenished every 3–4 days. Organoids were cultured for 7–9 days, and then passaged using Accumax. For stem cell media, advanced DMEM/F12 (Dulbecco’s modified Eagle’s medium/Nutrient Mixture F12) medium containing 1× HEPES, 1× glutamax, 1× N2 supplement, 1× B27 supplement, 1 mM *N*-acetyl-l-cysteine, 10 nM gastrin, and penicillin and streptomycin was prepared as base media. Then, 50 ng/mL recombinant epidermal growth factor, 100 ng/mL Noggin, 100 ng/mL Wnt 3A, 10 mM nicotinamide, 500 nM A83-01, 10 µM SB202190, and 100 ng/mL R-Spondin 2 were freshly added into the base media immediately before use. For histology, organoids were recovered from Matrigel with Gentle Cell Dissociation Reagent. After fixation in 4% paraformaldehyde at 4 °C overnight, organoids were embedded in Histogel Specimen Processing Gel, followed by general paraffin embedding and processing steps.

### PCR and real-time quantitative PCR

At 12 days postnatally, tail snips were collected from mice according to IACUC-approved procedures. The tails were incubated in 400 µL of 0.05 N NaOH at 95 °C for 1.5 h, briefly vortexed, and neutralized with 32 µL of 1 M Tris-HCl, pH 8. To genotype the mice used in this study, PCR analysis was performed using G-bioscience *Taq* Polymerase and included 10× buffer, along with dNTPs, RediLoad loading buffer (Invitrogen), and primers indicated by the Jackson Labs or NCI, using thermo-cycler parameters also indicated by the Jackson labs or NCI (Supplementary Table 1). Genotype for the Ptgs2-floxed allele was performed as described by the Herschman group^43^. PCR product was run on a 2% agarose gel containing ethidium bromide and was imaged with a BioRad ChemiDox XRS^+^. For RNA extraction and subsequent Ptgs2 expression analysis by real-time quantitative PCR (qRT-PCR)^54^, tissue was homogenized in Trizol using a handheld tissue homogenizer, and RNA was isolated by the manufacturer’s instructions. First-strand synthesis was performed using qScript cDNA Synthesis Kit (Quanta Bio), and qPCR was performed using SYBRgreen fluorophore along with forward and reverse primers on the BioRad CFX-96. Expression was normalized to β-actin^6^. All primers used for genotyping and qRT-PCR can be found in Supplementary Table 1.

### Statistical analysis

Pair-wise comparisons between two groups were analyzed by two-tailed Student’s *t* test. The statistical differences in tumor incidence between groups were determined by two-tailed, Fisher’s exact test. Statistical significances were considered as *p* < 0.05 (*); *p* < 0.005 (**); *p* < 0.0005 (***). Experimental data were demonstrated as the mean ± standard error of the mean, and sample numbers were described in each figure legend.

### Reporting summary

Further information on research design is available in the Nature Research Reporting Summary linked to this article.

## Supplementary information

Supplementary Information

Reporting Summary

Source Data

## Data Availability

The data supporting this study are available in the article or Supplementary Information or available from the authors upon reasonable request. The source data underlying Figs. 1f, 2h, 3d, 4c–e, g–j, 5c, e, h–k, and Supplementary Figs. 1c, 4b, d, 6d and 8a are provided as a Source Data File.
