# Supplementary material for: Krt5+/Krt15+ foregut basal progenitors give rise to cyclooxygenase-2-dependent tumours in response to gastric acid stress
Source: Nat Commun. 2019 May 20;10:2225. doi: 10.1038/s41467-019-10194-0 (PMC6527614; doi:10.1038/s41467-019-10194-0)
Supplement: Supplementary file 1 — Supplementary Information [file 41467_2019_10194_MOESM1_ESM.pdf]

SUPPLEMENTARY INFORMATION

Moon et al. $Krt5^+$/$Krt15^+$ foregut basal progenitors give rise to cyclooxygenase-2-dependent tumours in response to gastric acid stress

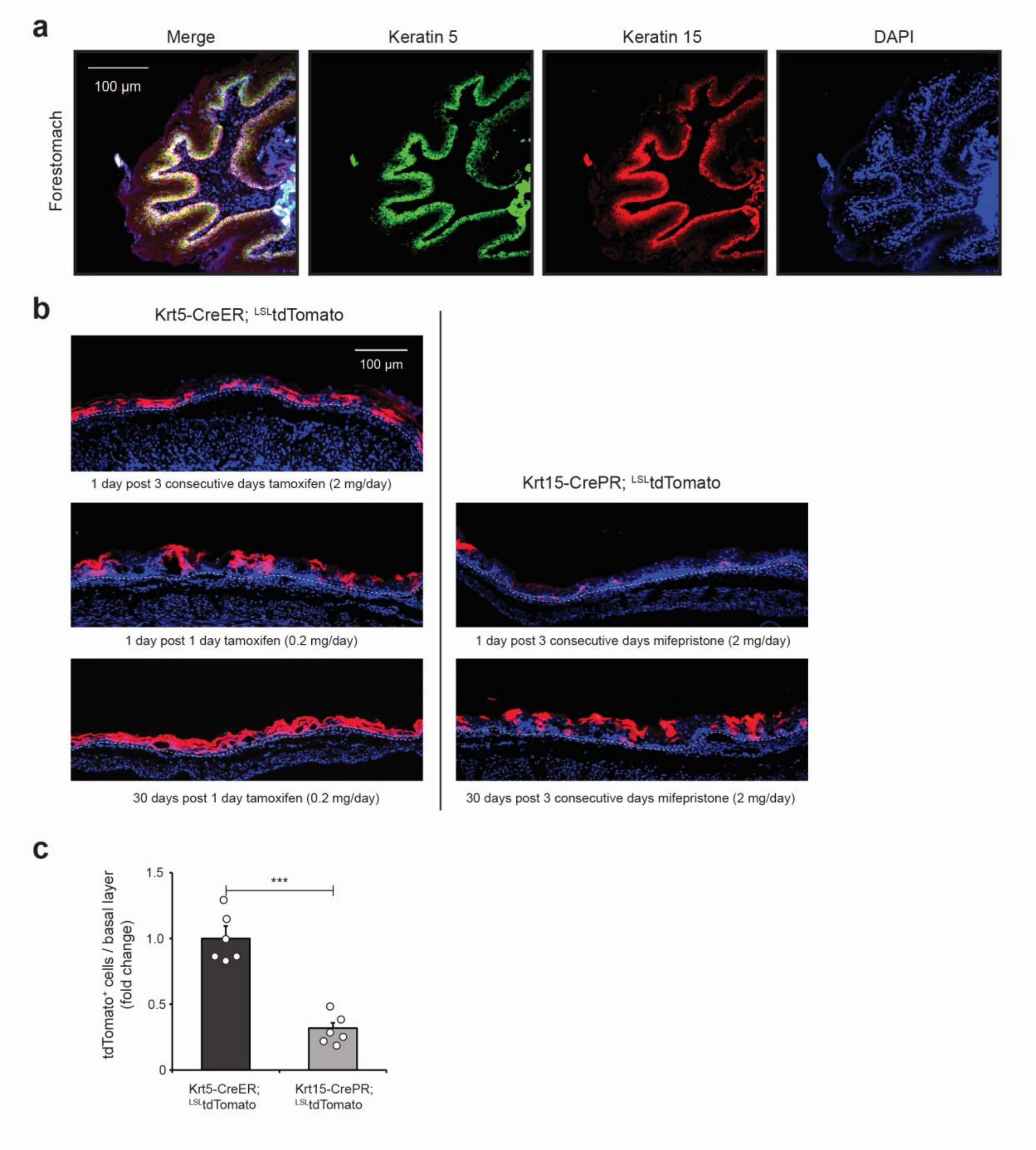

Supplementary Figure 1. Expression of Krt5 and Krt15, and genetic recombination of Krt5-CreER and Krt15-CrePR. (a) Immunofluorescence staining of Krt5 and Krt15 in murine forestomach tissues. (b) tdTomato expression induced by Krt5-CreER and Krt15-CrePR after tamoxifen (3 consecutive days, 2 mg i.p., and 1day, 0.2 mg i.p.) or mifepristone (RU486, 3 consecutive days, 2 mg i.p.) treatment. (c) Relative efficiency in labeling foregut basal progenitors between Krt5-CreER and Krt15-CrePR. n = 6 animals per group. Data are represented as mean ± SEM. Statistical significance, $^{***}p < 0.0005$. Scale bar, 100 μm.

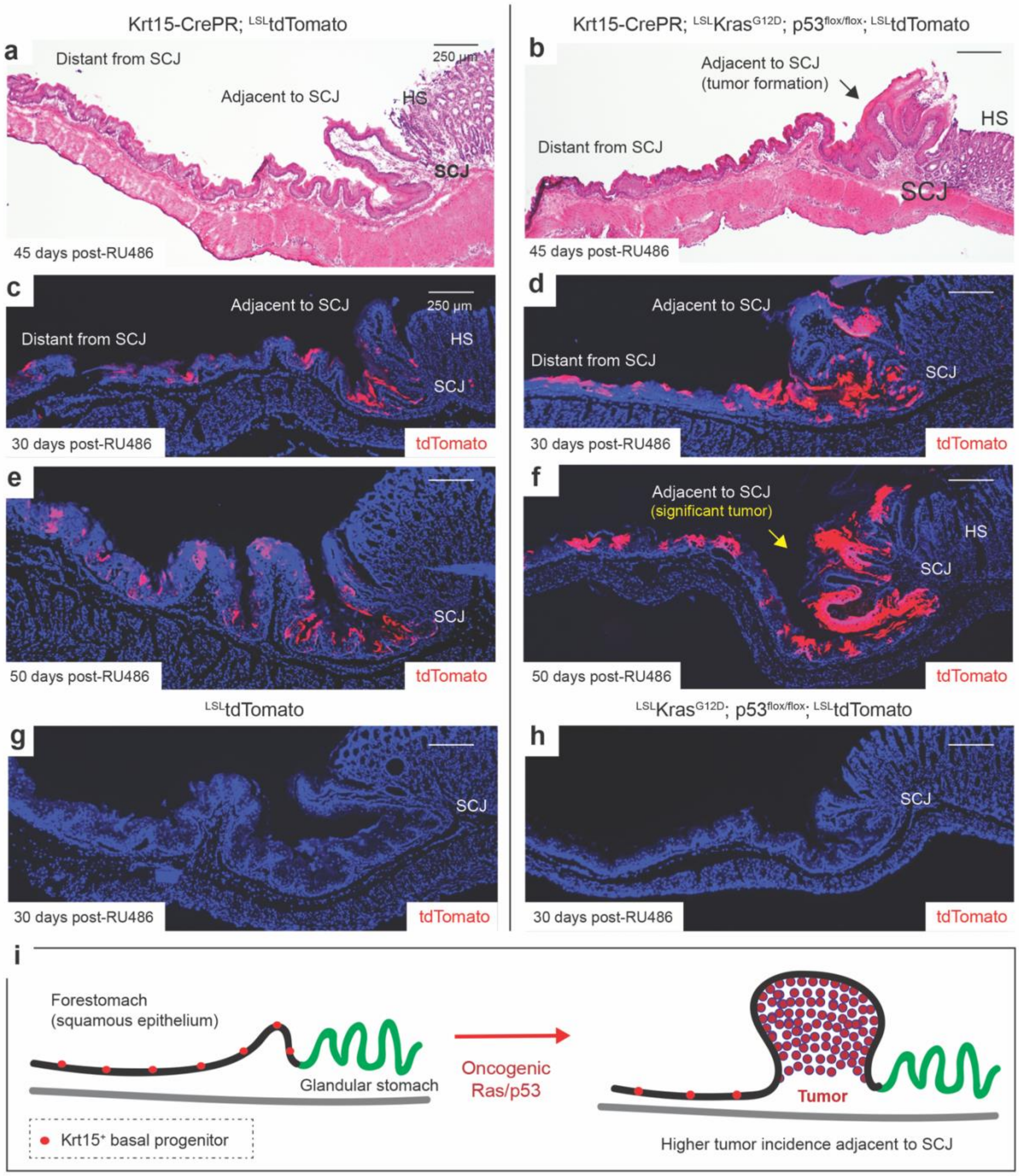

Supplementary Figure 2. Tumor-prone Krt15^{+} progenitors show the highest incidence of tumor initiation at the region adjacent to the SCJ in a Ras/p53 model. (a and b) Histology of forestomach tissues from Krt15-CrePR; LSL-tdTomato (control) and Krt15-CrePR; LSL-Kras^{G12D}; p53$^{flox/flox}$; LSL-tdTomato mice. (c-f) tdTomato lineage tracing demonstrated Ras/p53-mediated tumor formation at the region adjacent to SCJ. (g and h) Negative control for tdTomato lineage tracing and tumor formation using Cre negative mice. (i) Summary of specific locale of higher tumor incidence from tumor-competent Krt15^{+} progenitors.

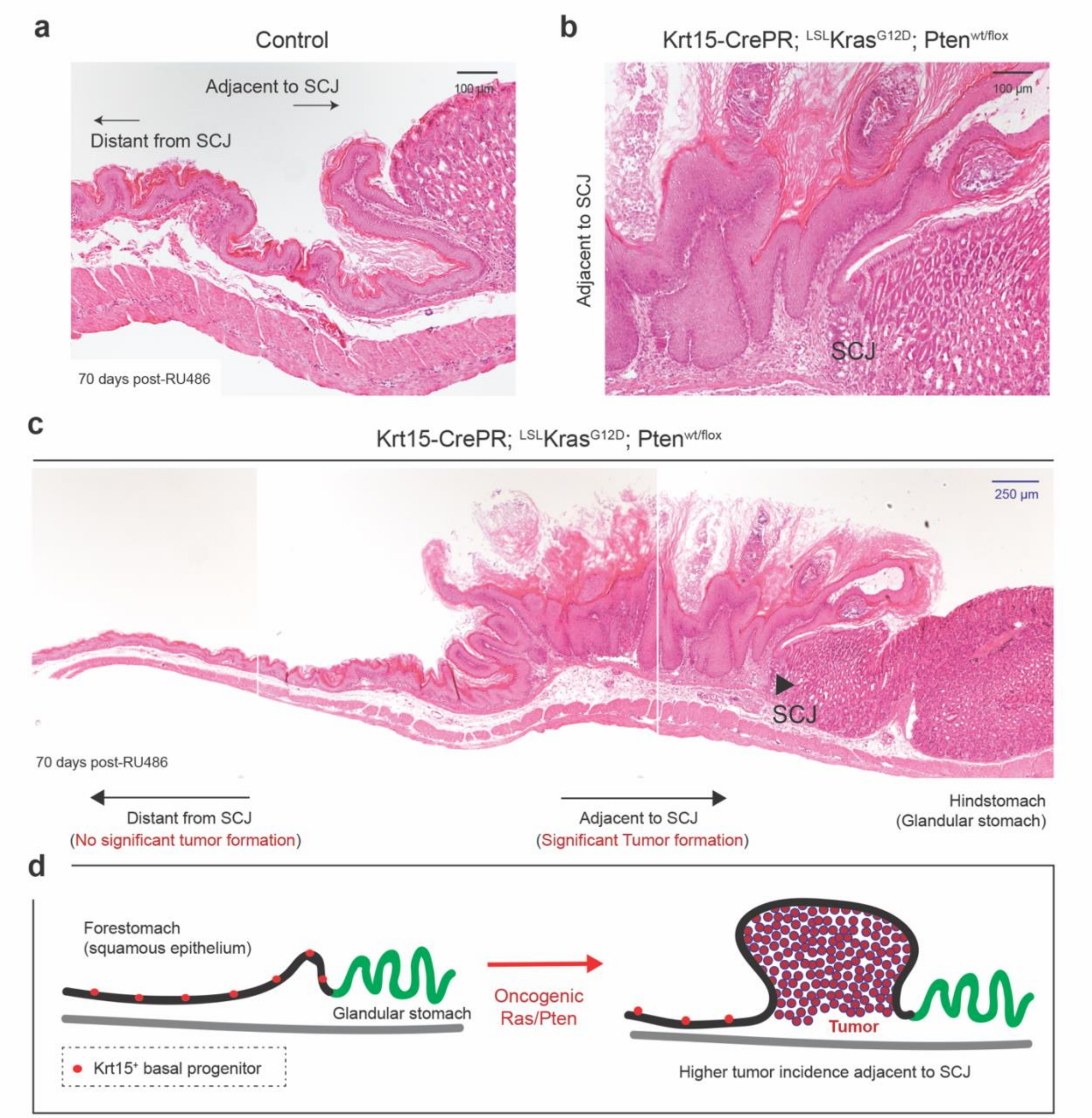

Supplementary Figure 3. Tumor formation preferentially occurs adjacent to the SCJ in a Ras/Pten model. (a-c) Histology of forestomach tissues from control and Krt15-CrePR; LSL-Kras^{G12D}; Pten$^{wt/flox}$ mice. A panoramic image was reconstructed by multiple images. (d) Summary of higher susceptibility of Ras/Pten-mediated tumor incidence adjacent to SCJ. Scale bar; 100 or 250 μm as indicated in each figure.

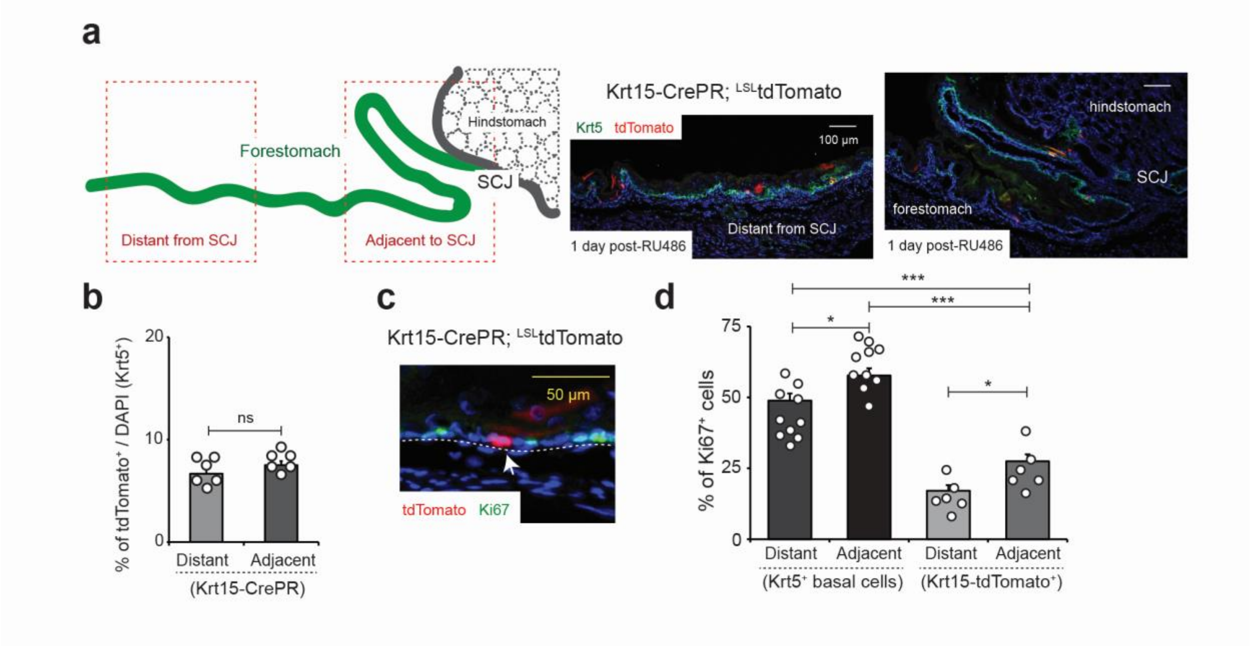

Supplementary Figure 4. Comparison of genetic recombination and proliferation rate. (a and b) tdTomato lineage tracing revealed similar genetic recombination between adjacent and distant regions, n = 6 animals. (c and d) Proliferation marker Ki-67 staining demonstrated higher proliferation at the region adjacent to the SCJ in basal cells (n = 10 animals) as well as the subpopulation of Krt15^{+} basal progenitors (n = 6 animals). Data are represented as mean ± SEM. Statistical significance, *$p < 0.05$, **$p < 0.005$, ***$p < 0.0005$, ns = not significant.

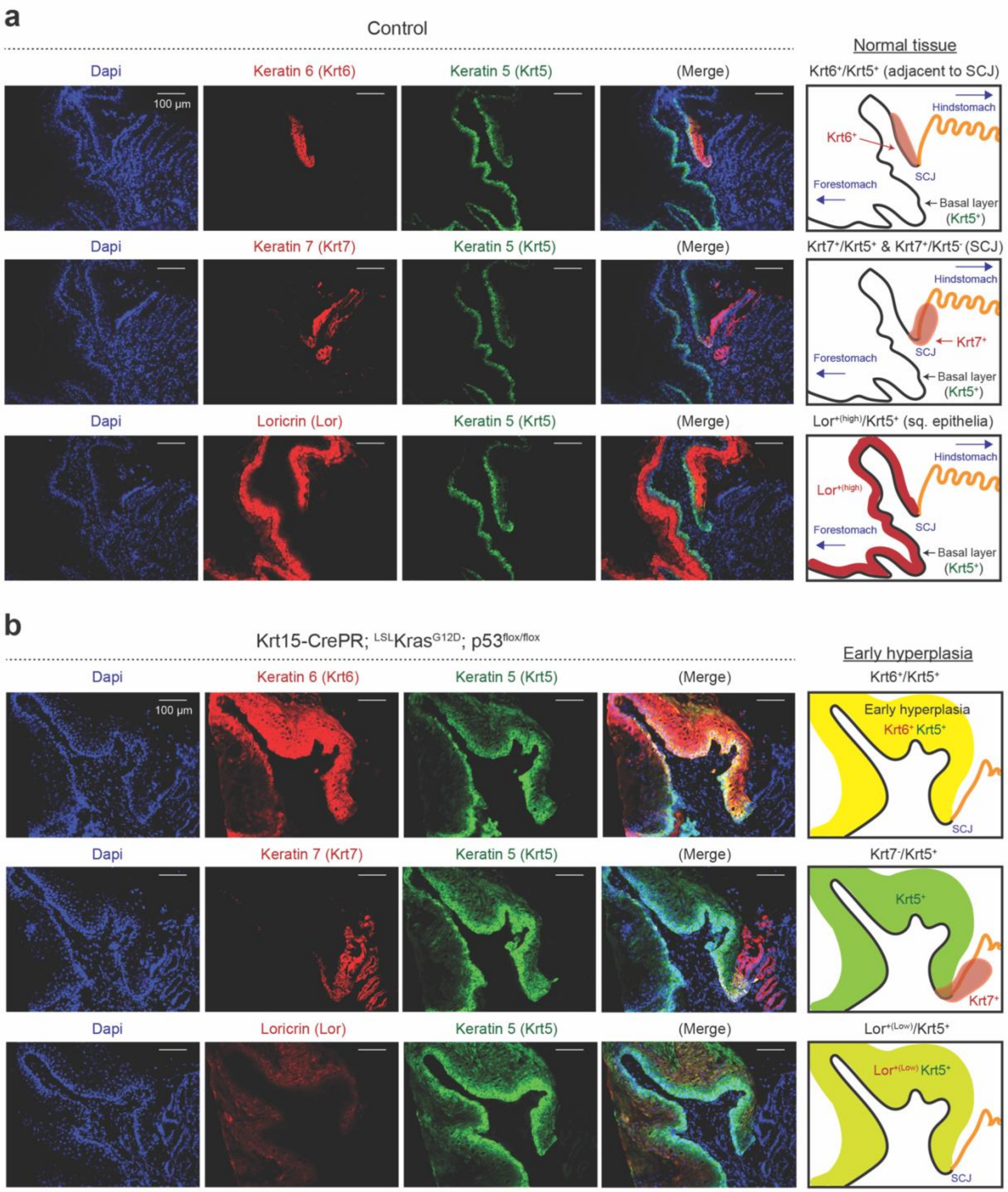

Supplementary Figure 5. Expression patterns of Krt5, Krt6, Krt7 and Lor during early tumor formation. (a) Krt5 and Loricrin (Lor) were used as markers for basal progenitors and differentiated epithelia in forestomach tissues, individually. Although generally absent throughout forestomach tissues, the expression of hyperplastic marker Krt6 was persistent in the forestomach region near the SCJ. At the SCJ,

Krt7/Krt5 double positive basal progenitors and $Krt7^+$ without Krt5 expression luminal cells were observed. (b) Early hyperplastic tumor cells from Krt15-CrePR; LSL-$Kras^{G12D}$; $p53^{flox/flox}$ mice showed high expression of both Krt5 and Krt6. Lor was weakly expressed in the hyperplastic tissues. Krt7 was not expressed in those tissues. Both Krt7 positive basal progenitors and luminal cells remain intact at the SCJ. SCJ, squamocolumnar junction; sq, squamous; Lor, Loricrin.

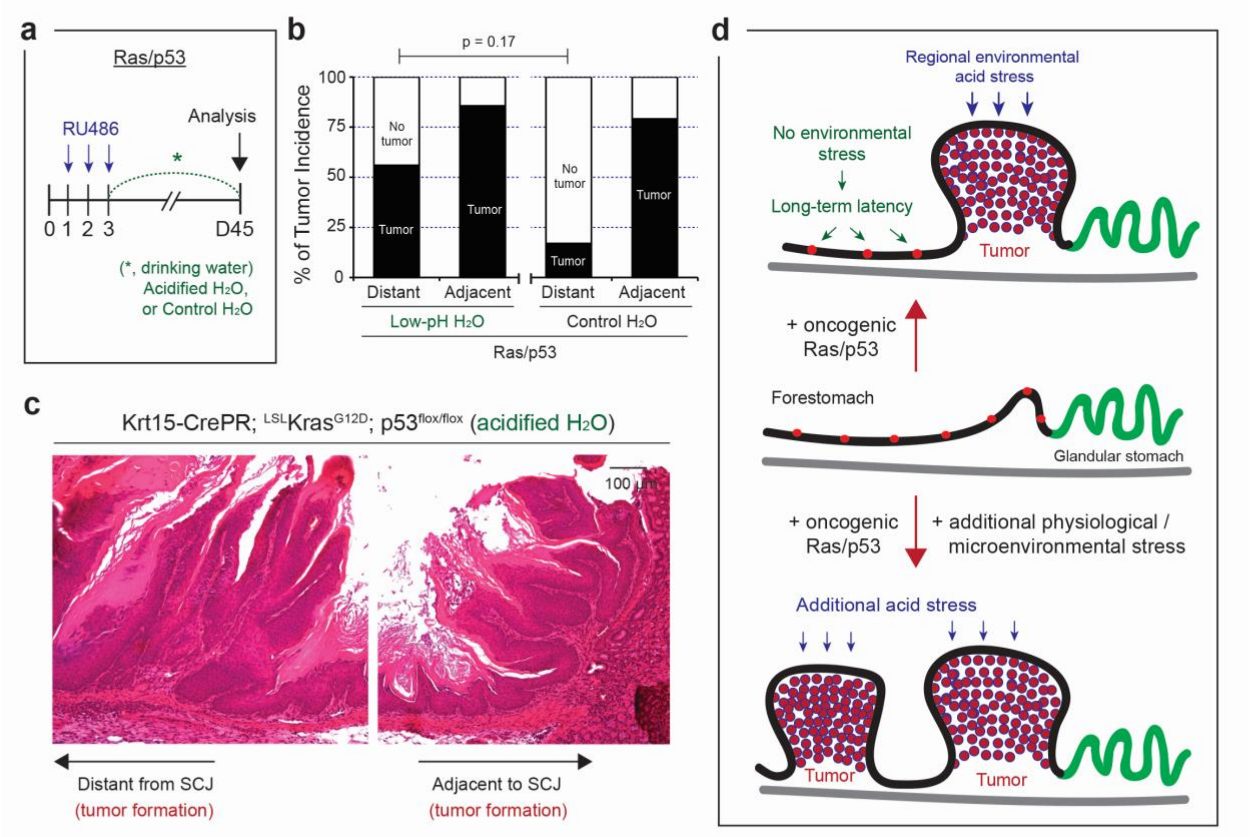

Supplementary Figure 6. Increased trend of tumor incidence by additional acidic stress. (a) Experimental scheme. (b) Ras/p53-mediated tumor incidence between groups with acidified and normal pH water, n = 9 animals per group. Statistical significance was determined using Fisher's exact test. (c) Histology demonstrated tumor formation from non-SCJ-adjacent region. (d) Summary of the results. Additional acid stress could increase the susceptibility of tumor formation from tumor-competent Krt15^{+} progenitors.

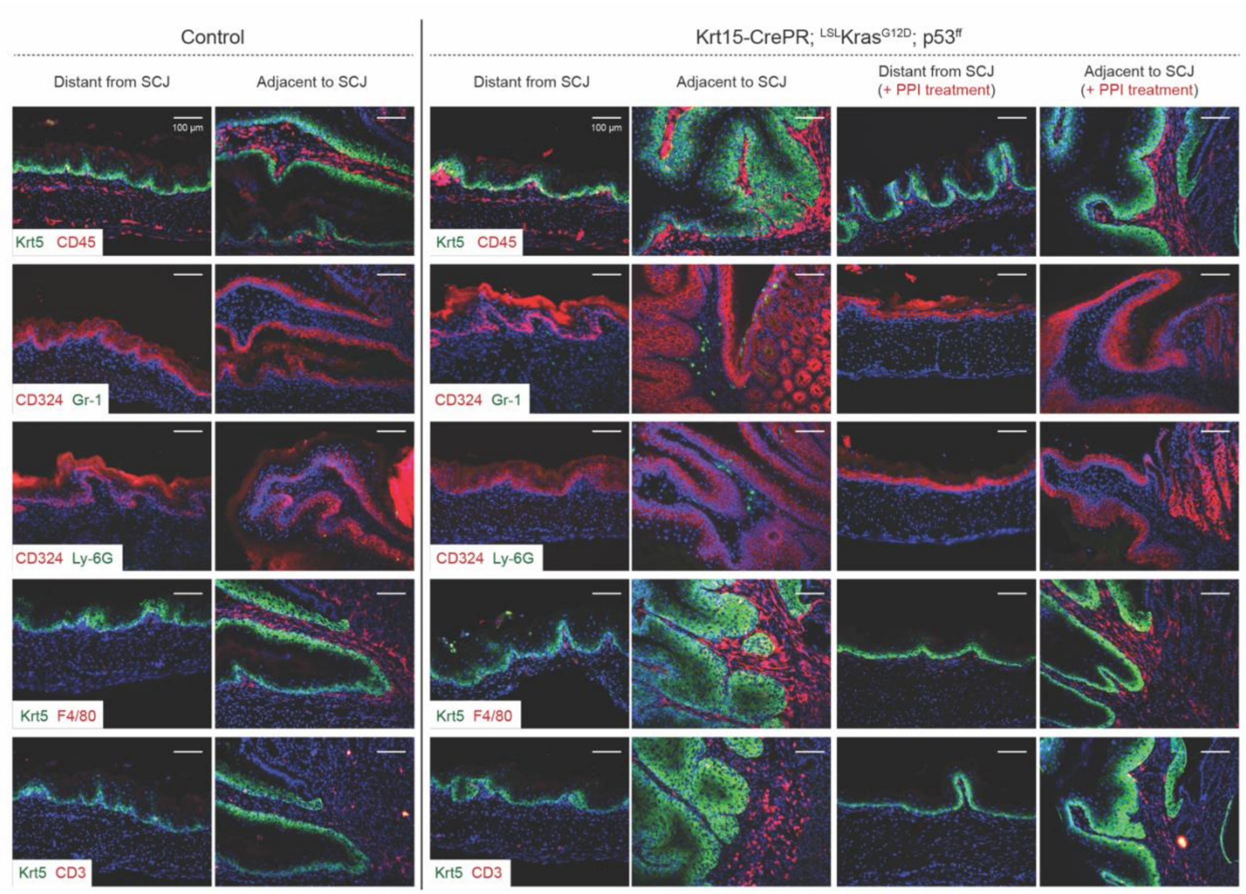

Supplementary Figure 7. Inflammation burden between locations and during tumor formation. Inflammatory burden was determined by inflammatory cell marker CD45, neutrophil markers Gr-1 and Ly-6G, macrophage marker F4/80 and T-cell marker CD3. Overall, higher inflammatory cells were present at the adjacent to SCJ in control, and the inflammatory burden significantly increased by tumor formation. PPI treatment suppressed tumor incidence rates together, and those tissues showed no abundant inflammation burden.

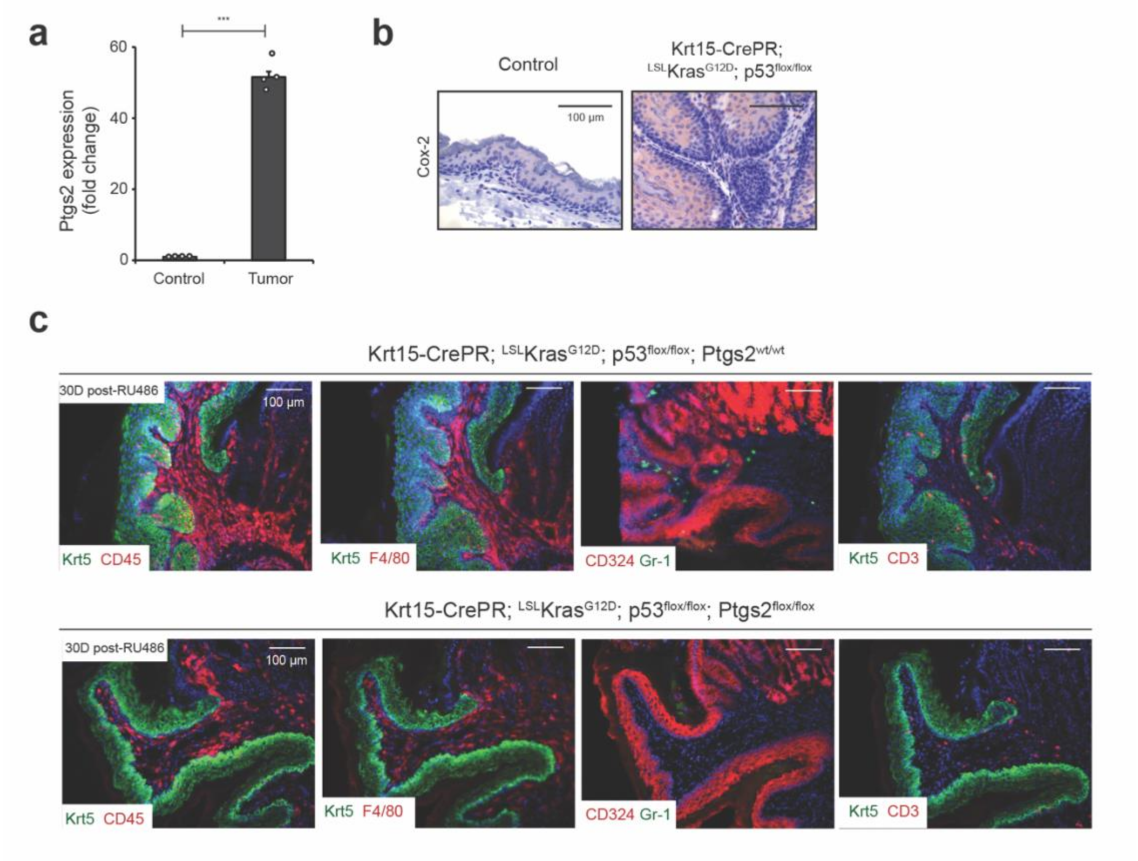

Supplementary Figure 8. Cox-2 expression and inflammation burden. (a) qRT-PCR showed significant upregulation of Ptgs2 expression in tumor tissues compared to healthy control (n = 4 independent replicates). Data are represented as mean ± SEM. Significance, ***$p < 0.0005$. (b) Cox-2 was significantly expressed in the tumor tissues from Krt15-CrePR; LSL-Kras^{G12D}; p53$^{flox/flox}$ mice. (c) Suppressed inflammatory burden was observed in Krt15-CrePR; LSL-Kras^{G12D}; p53$^{flox/flox}$; Ptgs2$^{flox/flox}$ mice, compared to Krt15-CrePR; LSL-Kras^{G12D}; p53$^{flox/flox}$; Ptgs2$^{wt/wt}$ mice.

Supplementary Table 1.

Gene of Interest	Primer Name	Sequence	Application	Source
Cre	cre3	GCA TTA CCG GTC GAT GCA ACG AGT GAT GAG	genotyping	Jackson Laboratories
	cre5	GAG TGA ACG AAC CTG GTC GAA ATC AGT GCG		
Kras	k005	AGC TAG CCA CCA TGG CTT GAG TAA GTC TGC A	genotyping	NCI Mouse Repository
	k006	CCT TTA CAA GCG CAC GCA GAC TGT AGA		
Pten	IMR 9554	CAA GCA CTC TGC GAA CTG AG	genotyping	Jackson Laboratories
	IMR 9555	AAG TTT TTG AAG GCA AGA TGC		
P53	IMR 8543	GGT TAA ACC CAG CTT GAC CA	genotyping	Jackson Laboratories
	IMR 8544	GGA GGC AGA GAC AGT TGG AG		
Ptgs2 (Cox2)	E3F1	AAT TAC TGC TGA AGC CCA CC	genotyping	Ishikawa and Herschman, 2006
	E4R1	CTT CCC AGC TTT TGT AAC CAT		
	E5F1	GTT CTT CTA CGG AGA GAG TTC		
	LoxR2	AGT GAA CCT CTT CGA GGG ACC		
	cox2-f	CAAGGGAGTCTGGAACATTG	qRT-PCR	Ghosh et al., 2006
	cox2-r	ACCCAGGTCCTCGCTTATGA		
tdTomato	TWTF	AAG GGA GCT GCA GTG GAG TA	genotyping	Jackson Laboratories
	TWTR	CCG AAA ATC TGT GGG AAG TC		
	TMF	CTG TTC CTG TAC GGC ATG G		
	TMR	GGC ATT AAA GCA GCG TAT CC		
β-actin	ActF	GATTACTGCTCTGGCTCCTAGC	qRT-PCR	
	ActR	GACTCATCGTACTCCTGCTTGC		

Supplementary References

1. Ishikawa, T.-O. & Herschman, H. R. Conditional knockout mouse for tissue-specific disruption of the cyclooxygenase-2 (Cox-2) gene. Genesis 44, 143–149 (2006).

2. Ghosh, S. et al. Essential role of tuberous sclerosis genes TSC1 and TSC2 in NF-kappaB activation and cell survival. Cancer Cell 10, 215–226 (2006).
