# Supplementary material for: Krt5+/Krt15+ foregut basal progenitors give rise to cyclooxygenase-2-dependent tumours in response to gastric acid stress
Source: Nat Commun. 2019 May 20;10:2225. doi: 10.1038/s41467-019-10194-0 (PMC6527614; doi:10.1038/s41467-019-10194-0)
Supplement: Supplementary file 2 — Reporting Summary [file 41467_2019_10194_MOESM2_ESM.pdf]

natureresearch

Corresponding author(s): Andrew C. White

Reporting Summary

Nature Research wishes to improve the reproducibility of the work that we publish. This form provides structure for consistency and transparency in reporting. For further information on Nature Research policies, see Authors & Referees and the Editorial Policy Checklist.

Statistical parameters

When statistical analyses are reported, confirm that the following items are present in the relevant location (e.g. figure legend, table legend, main text, or Methods section).

n/a	Confirmed	
☐	☒	The exact sample size (*n*) for each experimental group/condition, given as a discrete number and unit of measurement
☐	☒	An indication of whether measurements were taken from distinct samples or whether the same sample was measured repeatedly
☐	☒	The statistical test(s) used AND whether they are one- or two-sided<br>*Only common tests should be described solely by name; describe more complex techniques in the Methods section.*
☒	☐	A description of all covariates tested
☐	☒	A description of any assumptions or corrections, such as tests of normality and adjustment for multiple comparisons
☐	☒	A full description of the statistics including central tendency (e.g. means) or other basic estimates (e.g. regression coefficient) AND variation (e.g. standard deviation) or associated estimates of uncertainty (e.g. confidence intervals)
☐	☒	For null hypothesis testing, the test statistic (e.g. *F*, *t*, *r*) with confidence intervals, effect sizes, degrees of freedom and *P* value noted<br>*Give P values as exact values whenever suitable.*
☒	☐	For Bayesian analysis, information on the choice of priors and Markov chain Monte Carlo settings
☒	☐	For hierarchical and complex designs, identification of the appropriate level for tests and full reporting of outcomes
☒	☐	Estimates of effect sizes (e.g. Cohen's *d*, Pearson's *r*), indicating how they were calculated
☐	☒	Clearly defined error bars<br>*State explicitly what error bars represent (e.g. SD, SE, CI)*

Our web collection on statistics for biologists may be useful.

Software and code

Policy information about availability of computer code

Data collection	*Provide a description of all commercial, open source and custom code used to collect the data in this study, specifying the version used OR state that no software was used.*
Data analysis	Graphpad Prism 6

For manuscripts utilizing custom algorithms or software that are central to the research but not yet described in published literature, software must be made available to editors/reviewers upon request. We strongly encourage code deposition in a community repository (e.g. GitHub). See the Nature Research guidelines for submitting code & software for further information.

Data

Policy information about availability of data

All manuscripts must include a data availability statement. This statement should provide the following information, where applicable:

- Accession codes, unique identifiers, or web links for publicly available datasets
- A list of figures that have associated raw data
- A description of any restrictions on data availability

Resource Data was provided. All data related to figures are available from the corresponding author on reasonable request

Field-specific reporting

Please select the best fit for your research. If you are not sure, read the appropriate sections before making your selection.

☒ Life sciences ☐ Behavioural & social sciences ☐ Ecological, evolutionary & environmental sciences

For a reference copy of the document with all sections, see nature.com/authors/policies/ReportingSummary-flat.pdf

Life sciences study design

All studies must disclose on these points even when the disclosure is negative.

Sample size	No sample-size calculation was performed. A minimum of 3 male and 3 females was used in any in vivo studies.
Data exclusions	No data was excluded from analysis for any reason other than being unusable.
Replication	No attempts at replication of experiments in the paper failed. Data in this paper was reproducible: numbers of experiments and source data has been provided.
Randomization	Animals and in vitro cultures were paired and distributed into control and experimental groups for side-by-side comparison. No formal allocation/ randomization was done; animals were taken as they became available.
Blinding	Animals and in vitro cultures were paired and distributed into control and experimental groups for side-by-side comparison. No formal allocation/ randomization was done; animals were taken as they became available.

Reporting for specific materials, systems and methods

Materials & experimental systems

n/a	Involved in the study
☒	☐ Unique biological materials
☐	☒ Antibodies
☐	☒ Eukaryotic cell lines
☒	☐ Palaeontology
☐	☒ Animals and other organisms
☒	☐ Human research participants

Methods

n/a	Involved in the study
☒	☐ ChIP-seq
☒	☐ Flow cytometry
☒	☐ MRI-based neuroimaging

Antibodies

Antibodies used	Ki67, eBioscience, 14-5698, SolA15<br>pH3, Cell Signaling, 3377, D3C9<br>Cox2, Cayman, 160106, aa 570-598<br>Krt5, Biolegend, PRB-160P, Poly19055<br>Krt5, Biolegend, SIG-3475, Poly9059<br>Krt6, Biolegend, PRB-169P, Poly19057<br>Krt7, Abcam, ab181598, EPR17078<br>Krt15, Biolegend, 833901, Poly18339<br>CD324, Biolegend, 147306, DECMA-1<br>CD45, Biolegend, 103101, 30-F11<br>Gr1, Biolegend, 108417, RB6-8C5<br>Ly6G, Biolegend, 127625, 1A8<br>F4/80, Biolegend, 123101, BM8<br>CD3, Biolegend, 100201, 17A2
Validation	All antibody information has been provided. For histology, immunofluorescence, and western blotting positive and negative control samples were used to confirm specificity and validated in the species being tested.

Eukaryotic cell lines

Policy information about cell lines

Cell line source(s)	All primary cells were directly collected from mice right before our in vitro experiments.
Authentication	None of cells used were authenticated.
Mycoplasma contamination	Mouse primary cells were not tested for mycoplasma.
Commonly misidentified lines (See ICLAC register)	*Name any commonly misidentified cell lines used in the study and provide a rationale for their use.*

Animals and other organisms

Policy information about studies involving animals; ARRIVE guidelines recommended for reporting animal research

Laboratory animals	Mice used in this study were acquired from Jackson Labs or the National Cancer Institute Mouse Models of Human Cancers Consortium repository. For conditional knock-in/out experiments, 6 to 8 week-old animals were used. No formal randomization was performed for animal studies, animals were used as they came available, paired with littermate controls where possible, there was no preference for male or female animals, both genders were included in each analysis.
Wild animals	*Provide details on animals observed in or captured in the field; report species, sex and age where possible. Describe how animals were caught and transported and what happened to captive animals after the study (if killed, explain why and describe method; if released, say where and when) OR state that the study did not involve wild animals.*
Field-collected samples	*For laboratory work with field-collected samples, describe all relevant parameters such as housing, maintenance, temperature, photoperiod and end-of-experiment protocol OR state that the study did not involve samples collected from the field.*
