# Supplementary material for: Krt5+/Krt15+ foregut basal progenitors give rise to cyclooxygenase-2-dependent tumours in response to gastric acid stress
Source: Nat Commun. 2019 May 20;10:2225. doi: 10.1038/s41467-019-10194-0 (PMC6527614; doi:10.1038/s41467-019-10194-0)

Source Data File

Fig. 1f related

Control

		Tumor		
		Absent	Present	Total
Tissue Region	Distant	8	0	8
	Adjacent	8	0	8
	Total	16	0	16

Distant, distant from the SCJ; Adjacent, adjacent to the SCJ

Krt5-CreER; LSL-Kras^{G12D}; p53$^{wt/flox}$; LSL-tdTomato

		Tumor		
		Absent	Present	Total
Tissue Region	Distant	6	2	8
	Adjacent	0	8	8
	Total	6	10	16

Distant, distant from the SCJ; Adjacent, adjacent to the SCJ

Source Data File

Fig. 2h related

Control **(80 days post-RU486)**

		Tumor		
		Absent	Present	Total
Tissue Region	Distant	9	0	9
	Adjacent	9	0	9
	Total	18	0	18

Distant, distant from the SCJ; Adjacent, adjacent to the SCJ

Krt15-CrePR; LSL-Kras^{G12D} **(50 days post-RU486)**

		Tumor		
		Absent	Present	Total
Tissue Region	Distant	8	1	9
	Adjacent	3	6	9
	Total	11	7	18

Distant, distant from the SCJ; Adjacent, adjacent to the SCJ

Krt15-CrePR; LSL-Kras^{G12D}; p53$^{flox/flox}$ **(45 days post-RU486)**

		Tumor		
		Absent	Present	Total
Tissue Region	Distant	7	2	9
	Adjacent	1	8	9
	Total	8	10	18

Distant, distant from the SCJ; Adjacent, adjacent to the SCJ

Krt15-CrePR; LSL-Kras^{G12D}; p53$^{flox/flox}$ **(80 days post-RU486)**

		Tumor		
		Absent	Present	Total
Tissue Region	Distant	6	2	8
	Adjacent	0	8	8
	Total	6	10	16

Distant, distant from the SCJ; Adjacent, adjacent to the SCJ

Source Data File

Fig. 3d related

Control

		Tumor		
		Absent	Present	Total
Tissue Region	Distant	7	0	7
	Adjacent	7	0	7
	Total	14	0	14

Distant, distant from the SCJ; Adjacent, adjacent to the SCJ

Krt15-CrePR; LSL-Kras^{G12D}; Pten$^{wt/flox}$

		Tumor		
		Absent	Present	Total
Tissue Region	Distant	6	2	8
	Adjacent	0	8	8
	Total	6	10	16

Distant, distant from the SCJ; Adjacent, adjacent to the SCJ

Source Data File

Fig. 4g related

Krt15-CrePR; LSL-Kras^{G12D}; p53$^{flox/flox}$ **(Vehicle control)**

		Tumor		
		Absent	Present	Total
Tissue Region	Distant	7	2	9
	Adjacent	1	8	9
	Total	8	10	18

Distant, distant from the SCJ; Adjacent, adjacent to the SCJ

Krt15-CrePR; LSL-Kras^{G12D}; p53$^{flox/flox}$ **(+PPI treatment)**

		Tumor		
		Absent	Present	Total
Tissue Region	Distant	9	0	9
	Adjacent	6	3	9
	Total	15	3	18

Distant, distant from the SCJ; Adjacent, adjacent to the SCJ

Source Data File

Fig. 5e related

Krt15-CrePR; LSL-Kras^{G12D}; p53$^{flox/flox}$; Ptgs2$^{wt/wt}$

		Tumor		
		Absent	Present	Total
Tissue Region	Distant	12	3	15
	Adjacent	4	11	15
	Total	16	14	30

Distant, distant from the SCJ; Adjacent, adjacent to the SCJ

Krt15-CrePR; LSL-Kras^{G12D}; p53$^{flox/flox}$; Ptgs2$^{flox/flox}$

		Tumor		
		Absent	Present	Total
Tissue Region	Distant	12	2	14
	Adjacent	10	4	14
	Total	22	6	28

Distant, distant from the SCJ; Adjacent, adjacent to the SCJ

Source Data File

Supplementary Fig. 6b related

Krt15-CrePR; LSL-Kras^{G12D}; p53$^{flox/flox}$ **(Low-pH H_2O)**

		Tumor		
		Absent	Present	Total
Tissue Region	Distant	4	5	9
	Adjacent	1	8	9
	Total	5	13	18

Distant, distant from the SCJ; Adjacent, adjacent to the SCJ

Krt15-CrePR; LSL-Kras^{G12D}; p53$^{flox/flox}$ **(Control H_2O)**

		Tumor		
		Absent	Present	Total
Tissue Region	Distant	6	3	9
	Adjacent	2	7	9
	Total	8	10	18

Distant, distant from the SCJ; Adjacent, adjacent to the SCJ

Source Data File

Figure 4h related (full blot images)

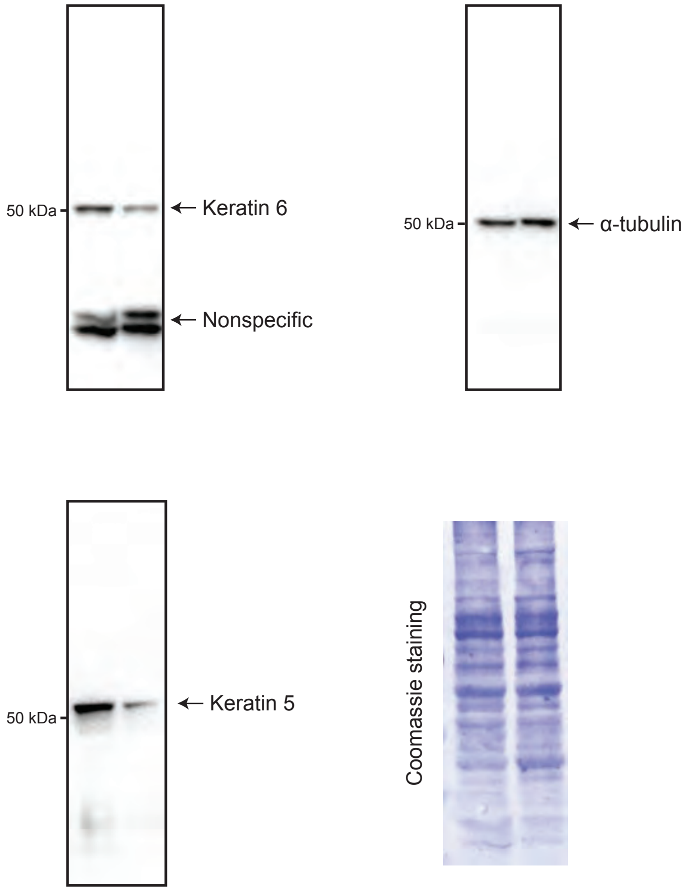

Source Data File

Figure 5h related (full blot images)

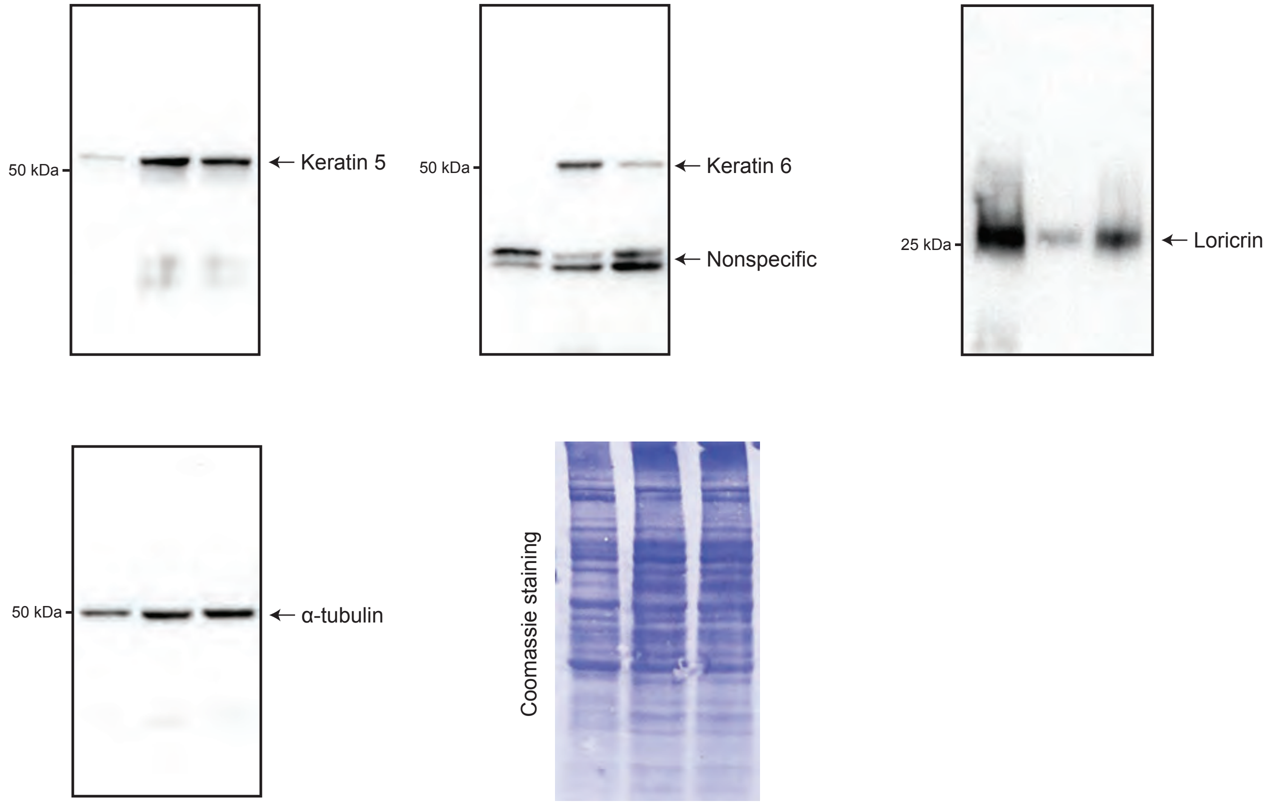

Supplement: Supplementary file 3 — Source Data [file 41467_2019_10194_MOESM3_ESM.zip › Source data.pdf]
